# Internal growth of women with recurrent miscarriage: a qualitative descriptive study based on the post-traumatic growth theory

**DOI:** 10.1186/s12905-023-02542-6

**Published:** 2023-07-21

**Authors:** Gisoo Shin, Hye Jin Kim, Sung Hae Kim

**Affiliations:** 1 College of Nursing, Chung - Ang University, 84 Heukseok-Ro, Dongjak-Gu, Seoul, 06974 Korea; 2 Department of Nursing, Changshin University, 262, Paryong-Ro, MasanHoiwon-Gu, Changwon-Si, Gyeongsangnam-Do, 51352 Korea; 3 Department of Nursing, College of Health, Welfare and Education, Tongmyong University, 428 Sinseon-Ro, Nam-Gu, Busan, 48520 Korea

**Keywords:** Internal Growth, Recurrent Miscarriage, Post-traumatic Growth, Women

## Abstract

**Background:**

Unexpected and repeated miscarriages in a woman's life cycle can be a mental and physical burden and lead to post-traumatic stress disorder. However, women may also experience inner growth with such experiences.

**Methods:**

This study was qualitative descriptive research examining the experiences of South Korean women who had recurrent miscarriages three or more times.

**Results:**

The average age of the participants was 34.6 years, and the average number of recurrent miscarriages was 3.87. Additionally, five themes were identified as follows: (1) Confusing as if in muddy water, (2) Self-examination of past daily life, (3) Empathy and comfort in homogeneous groups, (4) Religious beliefs that include the lost fetus, and (5) Transforming for internal growth.

**Conclusion:**

Based on the results of this study, intervention strategies need to be implemented to support the inner growth of women who have experienced recurrent miscarriages.

**Supplementary Information:**

The online version contains supplementary material available at 10.1186/s12905-023-02542-6.

## Background

The experience of losing someone in a woman's life cycle can vary. The process of becoming a mother through childbirth, which involves sharing life with a loved one, carries the meaning of family formation and being together for a lifetime [1]. However, women often experience a sense of loss due to unintentional miscarriage [2]. The birth rate in South Korea has been continuously decreasing since 2016, and as of 2022, it stands at 0.77, which is the lowest in the world; moreover, the rate of miscarriages among women is increasing, making it a national issue in South Korea [3].

A miscarriage refers to the loss of the fetus within 20 weeks of pregnancy, and chromosomal abnormalities of the fetus are a known major cause. Nevertheless, it can also occur without a clear cause or can occur due to various environmental factors [4]. When miscarriages occur repeatedly more than three times, they are defined as recurrent miscarriages (RMs), and women who have experienced RMs lose confidence in their own bodies and have negative emotions such as stress, anxiety, and confusion [5]. Sometimes, women who have experienced RMs may also show an obsession with the deceased fetus, or they may have difficulty coping with daily life due to guilt [6]. The sadness of women who have experienced RMs is difficult to comprehend, and in some cases, it can lead to life-threatening trauma [7, 8]. Post-traumatic stress disorder (PTSD) can arise as a reaction to experiencing a real or perceived threat of death or injury to oneself or others, and the experience of RMs can be shocking enough to cause PTSD [9].

Nevertheless, it has been reported that women who have experienced RMs can sometimes transcend their pain and gain positive changes, especially an increase in empathy, the ability to develop closer relationships with others, and a greater sense of personal strength and growth [10]. This can be explained by the adaptation, adjustment, and transformation that occur in response to a significant and unexpected loss in a woman's life, which are collectively known as post-traumatic growth (PTG). The concept of PTG was introduced by Tedeschi and Calhoun [11]. Traumatic events allow people to experience not only negative emotions but also internal adaptability and positive changes as individuals. Tedeschi and Calhoun [11, 12] described rumination as the cognitive processing of traumatic events, which seeks to find positive aspects in negative emotional situations and aims to reduce pain in a more goal-oriented manner. Rumination is divided into intrusive rumination and deliberate rumination, which are important factors contributing to PTG [11, 12]. The PTG theory suggests that deliberate rumination can lead to positive psychological changes and personal growth [12]. A RMs itself is a traumatic event that can cause intense emotions and distress; thus, women who have experienced RMs can find new meaning in their lives through the deliberate rumination of the traumatic experience [7].

**Fig.1:**
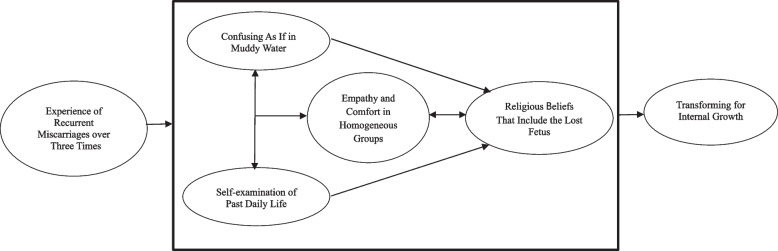
Concept framework of the present study based on the post-traumatic growth theory

Qualitative research methods have been applied to interpret and understand the phenomenon of RMs experienced by women. Among the various qualitative research methods, the qualitative descriptive (QD) method is chosen when seeking direct descriptions of who, what, and the phenomenon, event, or experience. One approach to QD analysis is thematic analysis, which involves identifying and analyzing recurring patterns through in-depth interviews with research participants [13]. Therefore, in this study, an approach based on the QD method was used to understand and analyze the experiences of South Korean women who had RMs three or more times (Fig. 1).

## Methods

### Research design

This study was qualitative research investigating the RMs experiences of women using a QD study method.

### Participants and recruitment

In this study, participants were recruited through convenience sampling among women who visited specialized hospitals for women located in Seoul and two provincial cities (Chungcheong Province and Gyeongsang Province). The qualitative research requires a minimum sample size of at least 12 to rich data saturation [14], thus a total of 30 participants were included following in-depth interviews in this study. However, 7 participants withdrew their consent to participate, resulting in a final sample of 23 individuals. The selection criteria for the participants in this study were as follows: over 19 years old, sufficient communication to participate in the study, and prior RMs experiences.

### Data collection and analysis

We assumed that the responses of the women to open-ended questions developed by the researchers could reflect their interpretation of RMs experiences (Additional file 1). In-depth interviews with the participants were conducted from May to July 2017, and data collection and analysis were carried out simultaneously. Data analysis was conducted until December of the same year after data collection was completed. Data collection was ended at a theoretical saturation point where the concept was judged to be rarely repeated or represented. The interview data were analyzed using a 6-step thematic analysis process proposed by Braun and Clarke [15]. The process was as follows: (1) Familiarizing yourself with the data: To understand the breadth and depth of the data, all public responses were repeatedly read by members of the research team. The team met several times to discuss initial impressions of the data. (2) Generating initial codes: All relevant phrases, sentences, or paragraphs related to the research question were extracted and given short labels or codes to capture the essence of the text unit. The team discussed, modified, and validated all codes. The codes were then organized into a table to present the data. (3) Searching for themes: Based on code similarity, the team divided the codes into comprehensive themes. Visual representations were generated to explore relationships between codes within and between themes. (4) Reviewing themes: Themes were reviewed and modified by the team and demonstrated consistent patterns. Consistent patterns included internal homogeneity (i.e., codes were meaningfully connected within each theme) and external heterogeneity (i.e., clear distinctions between themes). The team revisited the entire narrative dataset to ensure that all relevant data were captured in one of the themes. (5) Defining and naming themes: Each participant was identified by statements that captured distinct aspects of the RMS experiences. (6) Producing the report: A final report was prepared, providing detailed descriptions for each theme. In addition, a qualitative data analysis software program (QSR Nvivo 10.0) was used by three researchers for coding, examining coding frequencies, identifying patterns, and extracting themes. Two team members (SH and G) performed the initial analysis, and peer reports were used at multiple stages of the process, with a third team member (HJ) independently verifying the conclusions through data re-examination. Additionally, one team member maintained an audit trail to document all methodology and analysis decisions, which was regularly reviewed by the other team members.

### Strategies to ensure rigor

To enhance the reliability and validity of the data, four aspects of rigor were considered [16]. Feedback on the analysis results was obtained from the research participants through in-depth interviews, and the results were also presented to a professor who gives lectures on qualitative research in a graduate program for consultation and feedback, thereby increasing the credibility of the research findings. Detailed descriptions were provided regarding the research content, the role of the researchers, participant recruitment, data collection, and analysis methods to enhance transferability. The data analysis process was repeated multiple times until consistent research findings were obtained (intra-rater reliability), thus ensuring dependability. Furthermore, consultation was sought from a professor who primarily conducts qualitative research to improve confirmability. To ensure completeness in the reporting of the results, the Consolidated Criteria for Reporting Qualitative Research (COREQ) [17] were applied.

## Results

### Sample characteristics

The mean age of the study participants was 34.6 years (range 28 ~ 42), and the average number of RMs was 3.87 (range 3 ~ 10). Among the participants, 14 individuals (60.8%) were currently unemployed, and 16 individuals (69.6%) were educated at the college level or higher (Table 1).

**Table 1:** Characteristics of participants (*N* = 23)

Participant	Age (years)	Number of miscarriages	Job	Education level
1	32	3	No	Above college
2	34	3	Yes	High school
3	40	3	Yes	High school
4	36	5	No	Above college
5	42	3	No	Above college
6	41	3	No	Above college
7	28	3	Yes	Above college
8	30	3	No	High school
9	32	5	No	Above college
10	29	3	No	Above college
11	40	3	Yes	Above college
12	34	3	No	High school
13	36	4	Yes	Above college
14	35	3	No	Above college
15	28	3	Yes	High school
16	41	7	No	Above college
17	30	3	Yes	Above college
18	32	3	No	High school
19	33	5	Yes	Above college
20	39	3	No	Above college
21	33	3	Yes	Above college
22	31	5	No	High school
23	39	10	No	Above college

### Themes reflecting the interpretation of RMs experiences

Five themes related to the women’s interpretation of RMs experiences were identified. Each theme is described below (Fig. 1).

### Theme 1: Confusing as if in muddy water

The first theme (Confusing as if in muddy water) could describe the experiences of the participants following RMs. All participants tended to experience a stage of strong denial after each miscarriage, and they attempted to seek out other hospitals for a diagnosis of a healthy pregnancy. However, with each subsequent miscarriage, the participants experienced greater feelings of anger as their expectations for a successful pregnancy were not met. Over time, the participants reported that their degree of depressive mood worsened. Most of the participants felt defeated or guilty in isolation, which heightened their feelings of confusion and made it difficult to process their emotions.

*“I think the pregnancy test is wrong. Others can easily have a healthy baby, even when they do not want a baby. I wonder why this is happening only to me who really wants a child, and I am so angry.”*

*“I cried a lot and thought that I was wrong and that it was my fault. I had many self-defeating thoughts... It's like my womb was buried in the mud.”*

*“I felt like I was walking through a dark tunnel from which I could not easily get out.”*

### Theme 2: Self-examination of past daily life

The second theme was most frequently talked about by the participants, which was 'Self-examination of past daily life'. Participants looked back on their past and questioned habits and behaviors that they had unintentionally engaged in. They carefully analyzed potential factors contributing to their RMSs, such as their dietary habits, quality of sleep, and traumatic events experienced during pregnancy. Additionally, they reflected on stressful situations they had experienced after becoming pregnant. Furthermore, they attempted to determine if these daily habits and behaviors could have been a cause of their miscarriages.

*“I remembered all the fast food I had eaten, such as cup noodles and hamburgers, and wondered whether this behavior was a problem.”*

*“Since I was married at 40, I should have been more careful with my actions and more stress-free every moment.”*

*“I began to look back and reflect on whether I had done anything wrong that had harmed others in my life. I also examined all of my past actions to see if any of them had inadvertently caused any distress or harms to the fetus without my knowledge.”*

### Theme 3: Empathy and comfort in homogeneous groups

The third theme (Empathy and comfort in homogeneous groups) was related to forming new relationships through empathy, which began by obtaining information on miscarriage as well as reflecting on daily life. All of the research participants searched through the internet, where necessary information can be easily accessed. It was found that women who had been previously diagnosed with a lifestyle-related miscarriage or who were currently experiencing a miscarriage joined social media groups without hesitation. Above all, the participants showed a strong sense of empathy when finding and acquiring information from actual cases of RMs. Although there were no social relationships in the past, there was a commonality in miscarriage as the participants could empathize with each other, understand and encourage each other, and forms new social relationships voluntarily. Sometimes, they communicated more actively through social media than with people who have formed and maintained social relationships with them, and they tried to maintain a strong sense of bond with common interests. Moreover, they said that they could feel relief and comfort by expressing worries and anxieties on social media, which they could not express to their family.

*“After the third miscarriage, I was afraid that I would miscarry again; thus, I stayed in my house and quit my job. I spent most of my time on the bed for 6 months. I joined a peer group of people who experienced miscarriage through the internet and listened to various case stories. This has been a great comfort to me.”*

*“When I had a miscarriage, I saw my husband cry too. Knowing that my husband is also struggling, I could not easily tell my husband about my pain. However, I was able to fully express my feelings in an online social networking group. It also gave me hope because it was possible to obtain information on how pregnant women succeeded in giving birth.”*

*"The comfort of someone who experienced the same pain as me was more helpful than the friends who gave birth to a baby.”*

### Theme 4: Religious beliefs that include the lost fetus

The fourth theme (Religious beliefs that include the lost fetus) touched on prayers for aborted fetuses regardless of religion, acceptance of RMs, and resilience to hope for future possibilities. The participants were in a situation of constant stress and trauma because of repeated miscarriage experiences that they could not resolve on their own. They tried to alleviate psychological exhaustion by forming an attachment with the lost fetus. In addition, the participants tried to interpret the meaning of the lost fetus through faith with a focus on reincarnation and angels.

*“After the loss of my baby, I went to the [Buddhist] temple and gave a hundred-day prayer despite having no religion. I've been doing this type of ritual to lead the soul to heaven for 3 years. Even now, at the appropriate time, I pray and make an offering…”*

*“I placed the photo of the Three Goddess of Birth governing childbirth in the refrigerator. Sometimes, I felt like a stranger because of my unusual behavior... but, I had my hopes projected there.”*

*“No matter what, the lost fetus is my child. I still keep the pregnancy test results and early ultrasound pictures. I constantly think of the one I lost as an invisible angel always by my side.”*

### Theme 5: Transforming for internal growth

The participants did not wish to remain in pain following the loss of the fetus and sought to convert their trauma into inner growth. The fifth theme (Transforming for internal growth) could describe the motive for changes in the perspective on life as a woman. The participants were able to switch to a new perspective on women that was not previously considered. They adopted a transformative view of pregnancy and childbirth that was taken for granted previously as a woman. They became grateful for their lives and their mothers. Furthermore, they had a greater acceptance of other women such as unmarried women, divorced women, and transgenders. They said that they came to appreciate the preciousness of life again and were able to grow and cope better with the current situation.

*“I am now over 40 years old but I think I've just become an adult. Previously, I thought that pregnancy and childbirth were easy and natural for women. My mother looks great, and I am grateful for her... My point for now is that I am growing up.”*

*“The experience of miscarriage is like mud in water... I don't think it can be cured. Nevertheless, I think that the experience is meaningful. I became more grateful for my life and started to think about what I would do as a good woman.”*

*“Previously, I was not allowed to live a woman's life in different ways. However, I also live an unpredictable life... so it could be possible... There is not only one way in a woman's life...”*

## Discussion

One of the fundamental premises of positive psychology is that every individual ultimately seeks inner growth [18]. Similarly, the theory of PTG suggests that positive changes can occur following a traumatic event, leading to a new perspective on life [11, 12]. In this study, women who experienced RMs not only experienced the negative aspects of loss but also underwent a transformation that reminded them of the value of life following an introspection of their assumptions and beliefs.

A miscarriage is a shocking traumatic event that could disrupt lives [19, 20]. However, in South Korea's family-oriented society, the occurrence of a natural miscarriage causes not only sadness but also guilt as women have a greater responsibility. Therefore, RMs can lead to the social deprivation and isolation of women in South Korea [21]. Although the number of women experiencing RMs is increasing worldwide, it remains a trauma that is not easily resolved for women [22].

Most people living in modern societies use the internet and social network services (SNSs) almost every day. Likewise, the participants in this study used the internet and SNSs in their daily life. However, after RMs, their use of SNSs was focused on the theme of ‘Empathy and comfort in homogeneous groups’. Recently, SNS users are actively forming peer groups with similar hobbies or purposes, and social influence is also increasing in these groups [23]. Although the SNS activities of the participants in this study started with the purpose of obtaining information, they developed emotional empathy beyond the dimension of information sharing as they had experienced RMs. In the formation of social relationships, empathy is known to play a key role in the process of mutual self‐disclosure [24]. Self‐disclosure is more easily achieved in the same group or between groups with common elements, and it may serve as a positive buffer against stress or a negative psychological state [25].

The participants in this study continued to develop an attachment to the lost fetus. Attachment refers to an individual's tendency to seek intimacy with a person based on strong emotional bonds [26]. The participants believed that they could reunite with their lost fetus through a new birth. They found meaning in the experience of RMs under the theme of ‘Religious beliefs that include the lost fetus’, which is an important aspect of traditional Korean culture that goes beyond physical boundaries or actual evidence. In the context of the South Korean culture, babies play a major role in protecting the souls of ancestors and are seen as psychological symbols that connect generations. Giving birth in this culture is also linked to wealth and prosperity [27, 28].

PTG describes an individual's experience of recovering from trauma. In PTG, after a period of emotional distress, an individual not only returns to pre-traumatic function but also has an opportunity for personal development [11, 29]. Deliberate rumination is the cognitive processing of traumatic events emphasized in PTG [11, 12]. The results of this study demonstrated that women who experienced RMs could achieve internal growth and find new meaning in life through deliberate rumination. This observation is consistent with the findings of previous studies showing that resilience to traumatic experiences or traumatic events can lead to PTG, which can reduce negative emotions and provide a new perspective on life [30]. According to the PTG theory, questioning a traumatic event brings about positive changes in another dimension and promotes the attainment of wisdom with a new perspective on life [11, 12]. Instead of focusing on negative emotions associated with RMs, such as guilt, sadness, fear, and depression, women could gain a new perspective on life and consequently experience inner growth [6]. Importantly, RMs also changed the participants' view of life in this study. The findings provide evidence confirming that PTG can positively influence social relationships with others. Therefore, women suffering from a miscarriage can achieve growth through resilience by adopting rumination and intervention strategies, ultimately improving their quality of life with emotional support and education [31, 32].

This study has some limitations. First, as the results of this study were obtained from in‐depth interviews with women in South Korea, generalization to other populations is limited. Second, as questions that can clarify the relationship between fetal loss and PTG by taking into account the characteristics of the participants, such as marriage age, etiology of miscarriage, and infertility diagnosis, were not addressed when analyzing the results, it was not possible to analyze the results in detail. Therefore, it is necessary to compare and analyze the internal growth changes of women who experienced RMs by thoroughly examining various factors such as national, regional, and individual characteristics in the future.

## Conclusion

In this study, women who experienced more than three miscarriages had time to reflect on their confusing emotions, self-examine their past daily lives, and at the same time, experience empathy and find comfort through like-minded groups on social media. Despite the trauma of repeated miscarriages, they attempted to acquire a future-oriented perspective by relying on their religious faith in religion. Through deliberate rumination, the participants in this study consistently achieved personal growth and changed their perspective on life as women. Therefore, it is necessary to comprehensively examine the experience of RMs as an unexpected crisis in the lives of women and identify research strategies that can help promote women's health and well-being.

## Supplementary Information

**Additional file 1. **The interview questionnaire. Consolidated criteria for reporting qualitative studies (COREQ): 32-item checklist.

## Data Availability

The datasets used for analysis in this study are available from the corresponding author.

## Abbreviations

PTG: Post-Traumatic Growth
PTSD: Post-Traumatic Stress Disorder
RMs: Recurrent Miscarriages
SNS(s): Social Network Service(s)
QD: Qualitative Descriptive
