# Supplementary material for: Internal growth of women with recurrent miscarriage: a qualitative descriptive study based on the post-traumatic growth theory
Source: BMC Womens Health. 2023 Jul 21;23:386. doi: 10.1186/s12905-023-02542-6 (PMC10362550; doi:10.1186/s12905-023-02542-6)
Supplement: Supplementary file 1 — Additional file 1. The interview questionnaire. Consolidated criteria for reporting qualitative studies (COREQ): 32-item checklist. [file 12905_2023_2542_MOESM1_ESM.docx]

**Additional File 1.The interview questionnaire**

| “Have you experienced any changes in your daily life attitude after the repeated miscarriage?”  “What changes have you experienced in your relationship with your family after having a repeated miscarriage?”  “What changes have you had in your social interpersonal relationships after experiencing repeated miscarriages?”  “Have you experienced any changes in your life or interpersonal relationships as a woman after repeated miscarriages?” |
| --- |

**Additional File 2. Consolidated criteria for reporting qualitative studies (****COREQ): 32-item checklist**

Developed from: Tong A, Sainsbury P, Craig J. Consolidated criteria for reporting qualitative research (COREQ): a 32-item checklist for interviews and focus groups. International Journal for Quality in Health Care. 2017;19(6), 349-357.

| No. Item | Guide questions/description | Reported on Page # |
| --- | --- | --- |
| Domain 1: Research team and reflexivity | | |
| Personal Characteristics |  |  |
| 1. Interviewer/facilitator | Which author/s conducted the interview or focus group? | 4,5 |
| 2. Credentials | What were the researcher’s credentials? E.g. PhD, MD | 4 and title page |
| 3. Occupation | What was their occupation at the time of the study? | 4 and title page |
| 4. Gender | Was the researcher male or female? | 4 and title page |
| 5. Experience and training | What experience or training did the researcher have? | 4 |
| Relationship with participants |  |  |
| 6. Relationship established | Was a relationship established prior to study commencement? | 4 |
| 7. Participant knowledge of the interviewer | What did the participants know about the researcher? e.g. personal goals, reasons for doing the research | 3-5 |
| 8. Interviewer characteristics | What characteristics were reported about the interviewer/facilitator? e.g. Bias, assumptions, reasons and interests in the research topic | 3-5 |
| Domain 2: study design | | |
| Theoretical framework |  |  |
| 9. Methodological orientation and Theory | What methodological orientation was stated to underpin the study? e.g. grounded theory,  discourse analysis, ethnography, phenomenology, content analysis | 4 |
| Participant selection |  |  |
| 10. Sampling | How were participants selected? e.g. purposive, convenience, consecutive, snowball | 4,5 |
| 11. Method of approach | How were participants approached? e.g. face-to-face, telephone, mail, email | 4,5 |
| 12. Sample size | How many participants were in the study? | 4,5 |
| 13. Non-participation | How many people refused to participate or dropped out? Reasons? | NA |
| Setting |  |  |
| 14. Setting of data collection | Where was the data collected? e.g. home, clinic, workplace | 4,5 |
| 15. Presence of non-participants | Was anyone else present besides the participants and researchers? | NA |
| 16. Description of sample | What are the important characteristics of the sample? e.g. demographic data, date | 4,5 |
| Data collection |  |  |
| 17. Interview guide | Were questions, prompts, guides provided by the authors? Was it pilot tested? | 4,5 and Supplementary file 2, not pilot tested |
| 18. Repeat interviews | Were repeat interviews carried out? If yes, how many? | 4,5 |
| 19. Audio/visual recording | Did the research use audio or visual recording to collect the data? | 4,5 |
| 20. Field notes | Were field notes made during and/or after the interview or focus group? | 4,5 |
| 21. Duration | What was the duration of the interviews or focus group? | 4,5 |
| 22. Data saturation | Was data saturation discussed? | 4,5 |
| 23. Transcripts returned | Were transcripts returned to participants for comment and/or correction? | 4,5 |
| Domain 3: analysis and findings | | |
| Data analysis |  |  |
| 24. Number of data coders | How many data coders coded the data? | NA |
| 25. Description of the coding tree | Did authors provide a description of the coding tree? | NA |
| 26. Derivation of themes | Were themes identified in advance or derived from the data? | 5-9 |
| 27. Software | What software, if applicable, was used to manage the data? | 5 |
| 28. Participant checking | Did participants provide feedback on the findings? | 4,5 |
| Reporting |  |  |
| 29. Quotations presented | Were participant quotations presented to illustrate the themes / findings? Was each  quotation identified? e.g. participant number | 5-9 |
| 30. Data and findings consistent | Was there consistency between the data presented and the findings? | 5-9 |
| 31. Clarity of major themes | Were major themes clearly presented in the findings? | 5-9 and figure 1 |
| 32. Clarity of minor themes | Is there a description of diverse cases or discussion of minor themes? | 5-9 |
